# Structural Characterization of Porcine Adeno-Associated Virus Capsid Protein with Nuclear Trafficking Protein Importin Alpha Reveals a Bipartite Nuclear Localization Signal

**DOI:** 10.3390/v15020315

**Published:** 2023-01-23

**Authors:** Mikayla Hoad, Emily M. Cross, Camilla M. Donnelly, Subir Sarker, Justin A. Roby, Jade K. Forwood

**Affiliations:** 1 School of Dentistry and Medical Sciences, Charles Sturt University, Wagga Wagga, NSW 2678, Australia; 2 Department of Microbiology, Anatomy, Physiology and Pharmacology, School of Agriculture, Biomedicine and Environment, La Trobe University, Melbourne, VIC 3086, Australia

**Keywords:** Adeno-associated virus, importin alpha, nuclear localization, binding interface, structure

## Abstract

Adeno-associated viruses (AAV) are important vectors for gene therapy, and accordingly, many aspects of their cell transduction pathway have been well characterized. However, the specific mechanisms that AAV virions use to enter the host nucleus remain largely unresolved. We therefore aimed to reveal the interactions between the AAV Cap protein and the nuclear transport protein importin alpha (IMPα) at an atomic resolution. Herein we expanded upon our earlier research into the Cap nuclear localization signal (NLS) of a porcine AAV isolate, by examining the influence of upstream basic regions (BRs) towards IMPα binding. Using a high-resolution crystal structure, we identified that the IMPα binding determinants of the porcine AAV Cap comprise a bipartite NLS with an N-terminal BR binding at the minor site of IMPα, and the previously identified NLS motif binding at the major site. Quantitative assays showed a vast difference in binding affinity between the previously determined monopartite NLS, and bipartite NLS described in this study. Our results provide a detailed molecular view of the interaction between AAV capsids and the nuclear import receptor, and support the findings that AAV capsids enter the nucleus by binding the nuclear import adapter IMPα using the classical nuclear localization pathway.

## 1. Introduction

Adeno-associated viruses (AAVs) have become an intensively studied group of viruses due to their utility and status as the world’s top candidate for use as viral vectors for gene therapy [1]. As members of the family *Parvoviridae* and genus *Dependopavovirus* [2,3], these single-stranded DNA viruses are relatively small (~4.7 kilobases) with a simple linear genome encoding two primary genes, *Rep* and *Cap*, flanked by inverted terminal repeats [4] that form hairpin structures by self-annealing [5]. There are 13 recognized serotypes [6] and over 150 different variants of AAV, with many displaying specific tissue tropism [7] determined by the receptor binding of the AAV capsid with the cell surface [8]. Despite a high prevalence of infection among vertebrates (the human population has been shown to have seroprevalence ranging from 15–90% depending on the AAV serotype [9,10,11,12]), AAVs are not known to cause physical disease manifestations [13], and possess only a minimal capacity to invoke the immune response [14]. This can be attributed to the inability of AAVs to replicate in host cells without the co-infection with another helper virus [15,16], such as herpesviruses, adenoviruses, or papillomaviruses [11].

Due to their safety and simplicity, AAVs are prominent candidate vectors in the field of gene therapy. Furthermore, AAVs are able to be tailored for a wide range of therapeutic purposes [17]. For example, studies have used sequence comparisons of different AAVs to identify residues which may be responsible for tissue tropism, and engineered these residues from one AAV to another, with the resulting chimeras transducing certain tissues more efficiently [18,19]. Currently, there are well over 130 clinical trials underway using AAV vectors [20], and currently three registered and approved drugs for clinical use [21].

Despite the numerous advantages of using AAV vectors for gene therapy, there are a number of issues that need to be considered and/or overcome. One important consideration is the presence of pre-existing immunity within human populations. Although the magnitude of the immune response to AAV is relatively small during natural infection or gene therapy transduction (making the process of therapeutic treatment safe), specific antibodies are still raised that may neutralize the virion [9,10]. This does present an issue when patients display an anamnestic antibody response that may reduce the overall efficacy of an approved AAV vector. Currently, all AAV vectors used in clinical trials have been based on primate-infecting isolates; however, to minimize the drawbacks arising from pre-existing immunity, AAV vectors not naturally encountered within primate populations are being closely studied. Significant differences in capsid sequences likely result in low or no cross-reactivity to human anti-AAV antibodies [22,23]. Reduced neutralization would make a substantial difference in transduction efficiency and subsequent transgene expression, and it has been demonstrated that a porcine-derived AAV was not neutralized by immunoglobulin G from a pool made of over 10,000 human donors [24]. A study published in 2014 by the same group [25] found that not only could different porcine AAV strains transduce mouse tissue, but that they did so with varying degrees of specificity, indicating that a unique tissue tropism profile can also be observed in heterologous host organisms. If porcine AAVs are to be seriously considered as a human gene therapy vector, further understanding of their biological interactions is required, including the mechanism through which porcine AAVs migrate through the cell, and how they interact with human host proteins to achieve this.

All AAVs for which cellular entry has been investigated have been demonstrated to transit the cell via a similar pathway. The capsid attaches to primary and secondary receptors of the cell surface [8,26,27,28], and the virion is then internalized through the use of multiple endocytic routes [29,30,31]. The translocation of AAV capsids into the host nucleus is a crucial step in this pathway, however, little is known about the interactions between AAV capsids and host transport proteins that allow import to occur [32].

In the classical nuclear import pathway, the cellular transport protein importin-α (IMPα), binds to a cluster of basic residues within a cargo protein [33] known as the NLS [34]. The protein IMPβ then binds to the IBB domain of IMPα [35], and this heterotrimeric complex is then translocated through the nuclear pore complex and into the nucleus. Here, RanGTP then dissociates the complex [36], and IMPα and IMPβ are recycled back into the cytoplasm.

The *Cap* gene of AAV encodes three capsid proteins that share a C-terminal sequence and combine to form the shell-like outer structure of the virus [37,38]. VP1 is encoded by the entirety of the *Cap* gene, and as a result is the largest capsid protein containing both N-terminal and C-terminal domains [39,40]. VP2 is translated from an alternatively spliced mRNA, and though it is encoded in the same reading frame as VP1, it is smaller with translation initiating on a start codon ~137 residues downstream of the VP1 start site [39,40]. VP3 is similarly expressed from an alternate mRNA through leaky scanning and is the smallest capsid protein, only maintaining the C-terminal domain from residue ~200 onwards [39,40]. These three capsid isoforms interlock to form the AAV capsid in a ratio of 1:1:10 (VP1:VP2:VP3), with the N-terminus of VP1 and VP2 located as a flexible segment inside the capsid shell [41]. During endosomal escape, conformational changes occur. In particular, the N-terminal regions of VP1 and VP2 become externally exposed on capsids via extrusion through the virion pores [42,43].

Studies have identified four basic regions (BR) in the AAV VP1 sequence [44]. Basic regions 1 (BR1), 2 (BR2), and 3 (BR3) are located on the N-terminal domain whilst basic region 4 (BR4) is on the C-terminal domain [44]. VP1 contains all four of the basic regions, VP2 retains only BR2, BR3, and BR4, whilst the much shorter VP3 only contains BR4 [44]. Studies have shown that mutation to BR1 altered nuclear transduction [44,45], and mutation of BR2 and BR3 reduced AAV VP1/VP2 to transduce into the nucleus [46]. Interestingly, mutational studies of BR4 have shown this region to have no impact on nuclear import of the capsid protein [44,46], but that this region does contribute to virion assembly [46].

Despite evidence supporting that these BR1-3 act as NLSs, very little data are available to define the way AAV capsids interact with host nuclear import receptors. Similarly, no evidence has yet been reported that could determine whether there is any isotype specificity to be found in interactions between the AAV capsid and different host importin proteins. We recently reported that an AAV porcine strain (Po1) [24] contained a region of the N-terminal capsid domain that is able to act as an NLS [47]. It was revealed that this AAV Po1 capsid NLS bound to mouse IMPα2 (a homolog of human IMPα1), with a high-resolution crystal structure of the complex defining the specific residues directly involved in this interaction. This was the first time a structure had been obtained revealing an importin protein in complex with a suspected AAV capsid NLS.

**Figure 1:**
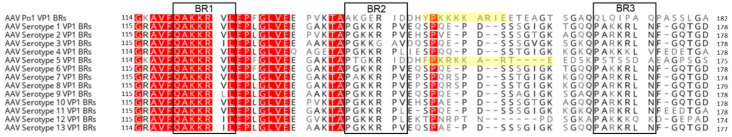
**Alignment of AAV VP1 N-terminal domain Basic Regions.** Alignment of human/primate AAV VP1 N-terminal domain encompassing basic regions 1–3 compared with AAV Po1 VP1 N-terminal domain. Sequences obtained from NCBI database: AAV Po1 (ACN42940.1), AAV 1 (NP_049542.1), AAV 2 (YP_680426.1), AAV 3 (NP_043941.1), AAV 4 (NP_044927.1), AAV 5 (YP_068409.1), AAV 6 (AAB95450.1), AAV 7 (YP_077178.1), AAV 8 (YP_077180.1), AAV 9 (AAS99264.1), AAV 10 (AAS99263), AAV 11 (AAT46339.1), AAV 12 (ABI16639.1), AAV 13 (ABZ10812.1). Residues highlighted in red show 100% sequence identity. Residues highlighted in yellow show a possible alternative basic region for AAV5 and AAV Po1. Geneious Prime (version 2020.2.3) was used to generate alignment.

Comparison of the sequence of AAV Po1 VP1 to primate AAV VP1 proteins revealed a similarity to human AAV5 (a shared amino acid similarity of ~85%) (Figure 1). Intriguingly, for AAV5 and AAV Po1 specifically, the positively charged cluster of amino acids comprising BR2 and BR3 are not congruent with the equivalent regions of the capsid amino acid sequence for the 12 other serotypes. Interestingly, AAV5 and AAV Po1 do not appear to hold a BR2 similar to that of the 12 other serotypes, and do not seem to contain any version of BR3 seen amongst the other serotypes. The region where BR2 would be expected to be observed on both AAV5 and Po1 lacks the prominent positively charged residues that form BR2. Instead, further downstream between where BR2 and BR3 would typically be found, is a region dense with positively charged residues (Figure 1). This overlapping region found on AAV5 and AAV Po1 is the suspected NLS sequence we have previously reported [47]; however, the homologous BR1 which was observed to be present in all the human AAV serotypes, and AAV Po1 was not investigated in our earlier study. Despite the BR1 sequence residing further upstream from the AAV5 and AAV Po1 BR2 than it does for other AAV serotypes, it remains possible that these two distinct basic regions may cooperate as a bipartite NLS during IMPα interactions.

Our present study reveals that the upstream BR1 region of the AAV Po1 capsid does indeed contribute to IMPα binding as part of an extensive bipartite NLS. A high-resolution structure demonstrates that BR1 (QAKKRVL^125^) interacts with IMPα at the minor cargo binding site, and the BR2-like NLS region (PKKKKAR^155^) we previously identified occupies the major site. Biochemical protein–protein interaction assays revealed that the bipartite AAV Po1 capsid NLS binds across all three IMPα subfamilies in preference to IMPβ. Furthermore, quantitative fluorescence polarization assays (FP) demonstrated a far superior binding strength of the bipartite NLS compared to the originally reported monopartite BR2-like region. These data allow insight into the mechanisms of AAV and IMPα binding, opening the potential for a much more direct targeting of capsid engineering. Evidently, understanding that bipartite NLSs are significantly superior in binding importin isoforms compared to monopartite NLSs will aid in the creation of an AAV capsid with an increased efficiency to translocate into a cell nucleus, and subsequently the overall effectiveness of AAV for gene therapy.

## 2. Materials and Methods

### 2.1. Gene Construct Design

The amino acid sequence of AAV Po1 VP1 protein was sourced through GenBank (accession ACN42940.1). A fragment of the VP1 gene corresponding to the amino acid residues ^114^ GKAVFQAKKRVLEPFGLVEEPVKTAAKGERIDDHYPKKKKARIEE TEAGTSGAQQLQIPAQP^175^ (referred to herein as AAV Po1 Cap-BR), was codon optimized for expression in *E. coli* and cloned into the pGEX4T-1 vector (GenScript, USA) at BamHI sites with an additional N-terminal tobacco etch virus (TEV) protease site for GST-tag cleavage. Mouse IMPα2 (mImpα2DIBB; His tag, no TEV site) was encoded by a pET30a expression vector as previously detailed [48].

For the importin proteins used for fluorescence polarization (FP) and electromobility shift (EMSA) assays, human importin α1 (hImpα1DIBB; His tag, TEV site), α3 (hImpα3DIBB; His tag, TEV site), α5 (hImp5DIBB; His tag, TEV site), α7 (hImpα7DIBB; His tag, TEV site), β (hImpβ; HIS tag, TEV site), and mouse importin α2 (mImpα2DIBB; His tag, no TEV site) were encoded by pET30a expression vector, and have been described previously [48,49,50].

### 2.2. Recombinant Expression and Purification

Expression and co-purification of AAV Po1 VP1 BR protein and IMPα2 was undertaken as described previously [47]. Overexpression of importin proteins α1, α2, α3, α5, α7, and β was performed in *E. coli* pLysS cells via the auto-induction method [51]. Post-induction, bacterial cells were pelleted via centrifugation at 6000 rpm for 20 min, and were subsequently resuspended using 20 mL per 2 L bacterial culture with HIS buffer A (50 mM phosphate buffer, 300 mM NaCl, 20 mM imidazole, pH 8), and lysed via two freeze-thaw cycles in addition to the application of 1 mL of 20 mg/mL lysozyme (Sigma-Aldrich, St. Louis, MI, USA), and 10 µL of 50 mg/mL DNase (Sigma-Aldrich, USA) at room temperature for 1 h. Soluble extract was harvested via centrifugation at 12,000 rpm for 30 min, and filtered through a 0.45 µm low protein affinity filter. Soluble extract was loaded and injected over a 5 mL HisTrap HP column (GE Healthcare, Chicago, IL, USA), and washed with twenty column volumes of His buffer A on an AKTApurifier FPLC (GE Healthcare, USA). An increasing concentration gradient of imidazole (20 mM to 500 mM) (ChemSupply, Gillman, SA, Australia) eluted the protein off the column, and resulting fractions were pooled. Size-exclusion chromatography was employed to further purify proteins using a HiLoad 26/60 Superdex 200 column (GE Healthcare, USA), pre-equilibrated in GST buffer A (50 mM Tris, 125 mM NaCl). Corresponding fractions of elution volume and protein size were collected and concentrated using Amicon MWCO 10 kDa filter (Merck Millipore, Burlington, MA, USA), and aliquoted for storage at −80 °C. Samples were assessed for purity by SDS-PAGE at 165 V for 30 min on a 4–12% Bis-Tris plus gel (Thermo Fisher Scientific, Waltham, MA, USA).

### 2.3. Crystallization of AAV Po1 Cap-BR and IMPα2 Complex

The protein complex was crystallized using the hanging drop vapour diffusion method with a final crystallization condition of 0.75 M Na Citrate, 0.1 M HEPES pH 6.5 mixed in a 1:1 ratio with a protein complex solution and crystallized at 22 °C. Rod-shaped crystals (100 × 10 × 10 μM) grew after 2 days of incubation. Crystals were collected and cryoprotected in the reservoir solution containing 20% glycerol, prior to being flash frozen to −196 °C in liquid nitrogen.

### 2.4. Data Collection and Structure Determination

X-ray diffraction data were obtained from the Australian Synchrotron on the MX2 beamline [52]. Data were indexed and merged through iMOSFLM [53], prior to scaling and subsequent merging via Aimless [54,55]. Phasing was undertaken using molecular replacement in Phaser [56], and the structure 4OIH from the Protein Data Bank (PDB) was used as a search model. Final model rebuilding and relative refining was performed in Coot [57,58,59] and Phenix [60], respectively.

### 2.5. Fluorescence Polarization Assay

Synthetic FITC tagged AAV Po1 capsid NLS peptides ^143^RIDDHYPKKKKARIEETEAGTSG^165^ (monopartite NLS) and ^119^QAKKRVLEPFGLVEEPVKTAAKGERIDDHYPKKKKARIEE^158^ (bipartite NLS; referred to as AAV Po1 Cap-BR) were obtained from GenScript. Peptides were incubated (2 nm) with two-fold serially diluted importin isoform (α1, α2, α3, α5, α7, and β) concentrations (starting concentration 4.5 μM) across 23 wells to a complete volume of 200 μL per well with GST buffer A (50 mM Tris, 125 mM NaCl). Fluorescence polarization measurements were recorded using a CLARIOstar Plus plate reader (BMG Labtech, Germany) in 96-well black Fluotrac microplates (Greiner Bio-One, Austria). Assays were performed in three independent experiments each containing an appropriate negative control (no importin binding partner). Data from the three independent experiments were assessed using GraphPad Prism (Prism 9, Version 9.3.1) and a binding curve fitted to determine the dissociation constant (K_D_).

### 2.6. Electro-Mobility Shift Assay (EMSA)

Synthetic FITC tagged AAV Po1 Cap-BR peptides (^119^QAKKRVLEPFGLVEEPVKTAAKGERIDDHYPKKKKARIEE^158^) obtained from GenScript were mixed with 20 μM of each importin isoform (α1, α2, α3, α5, α7, and β) supplemented with 3 μL 50% glycerol. Samples were run on a 1.5% agarose TB gel (3 mM TRIS, 1 mM Boric Acid, 1.5% agarose, pH 8.5) for 1.5 h at 70 V in TB running buffer (3 mM TRIS, 1 mM Boric Acid, pH 8.5). Images were recorded using a SYBR green filter of the GEL Doc BioRad Gel Doc imaging system. Gel was then stained using Coomassie stain for 10 min and destained with 10% ethanol and 10% glacial acetic acid overnight prior to imaging with Gel Doc BioRad Gel Doc imaging system.

## 3. Results

### 3.1. Purification of the IMPα2 and AAV Po1 Cap-BR Complex

**Figure 2:**
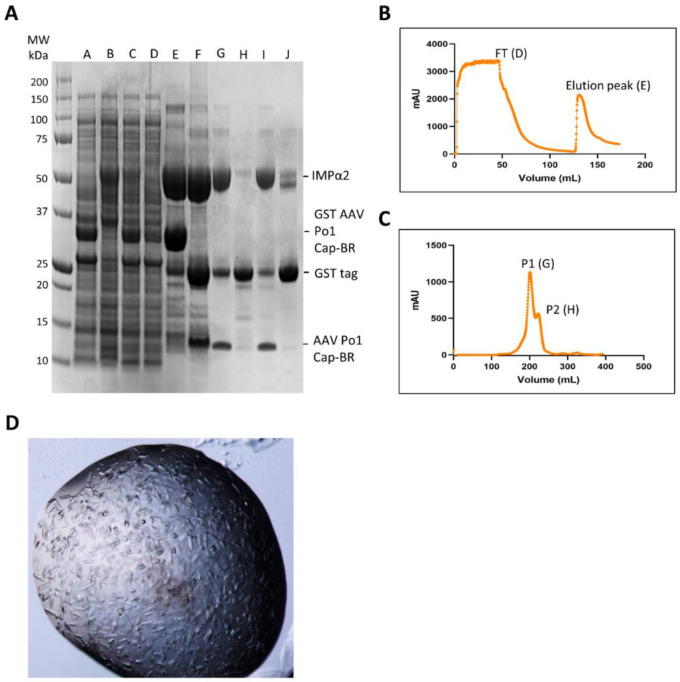
**Co-purification of AAV Po1 Cap-BR in complex with IMPα2.** (**A**) SDS-PAGE analysis of the purification process. Lane A: whole cell lysate of cells expressing GST-AAV Po1 Cap-BR. Lane B: whole cell lysate of cells expressing mouse His-IMPα2. Lane C: soluble extract of the combined whole cell lysates. Lane D: flow-through fraction from the Ni-affinity column. Lane E: Ni-affinity column elution demonstrating recovery of both His-IMPα2 and GST-AAV Po1 Cap-BR. Lane F: Ni-affinity column elution fraction post-digestion with TEV protease demonstrating cleavage of the GST tag. Lane G: elution fraction P1 from size-exclusion chromatography. Lane H: elution fraction P2 from size-exclusion chromatography. Lane I: flow-through fraction of the GST column demonstrating purified AAV Po1 Cap-BR:His-IMPα2 complex. Lane J: GST column elution. (**B**) UV trace of Ni-affinity co-purification of mouse His-IMPα2 with GST-AAV Po1 Cap-BR, with flow through and elution peaks indicated (with corresponding lanes from panel A in parentheses). (**C**) UV trace of size-exclusion chromatography of the TEV-cleaved His-IMPα2:AAV Po1 Cap-BR complex indicating elution peaks P1 and P2 (with corresponding lanes from panel A in parentheses). (**D**) Protein crystals of IMPα2 in complex with AAV Po1 Cap-BR. Rod-shaped crystals formed after 2 days of incubation at room temperature with 0.75 M Na Citrate, 0.1 M HEPES pH 6.5.

Our earlier structural study demonstrated the manner in which the AAV Po1 VP1 suspected monopartite NLS was able to bind with IMPα2, providing the first published data on the structural interactions between an AAV capsid and a host nuclear import protein [47]. Here we used a similar method to gather further data and structural evidence to determine IMPα2 binding in the context of a larger portion of the AAV Po1 VP1 containing residues that span the positions of all three N-terminal BRs. AAV Po1 Cap-BR and IMPα2 were individually expressed within *E. coli* cells prior to co-purification via Ni-affinity and size exclusion chromatography. Recombinantly expressed protein extracts were combined, and His-IMPα2 and GST-Po1 Cap-BR were observed to interact despite the presence of non-specific proteins within the *E. coli* lysate [Figure 2A,B]. Ni-affinity purification of *E. coli* lysate showed binding between IMPα2 and AAV Po1 Cap-BR in what appears to be a 1:1 ratio [Figure 2A,B]. The GST-affinity tag was cleaved from AAV Po1 Cap-BR using TEV proteolysis, and then the complex was further purified via size-exclusion chromatography [Figure 2A,C]. Removal of the remaining contaminating GST affinity tag was performed via injection of the size exclusion-purified sample over a glutathione column with collection and concentration of the column flow-through [Figure 2A]. Samples were taken at every stage and examined via SDS-PAGE analysis. The complex formed by AAV Po1 Cap-BR and IMPα2 was observed to be stable over multiple purification steps. The purified complex was concentrated to 34 mg/mL for use in crystallization trials.

### 3.2. Protein Crystallization and Data Collection

**Figure 3:**
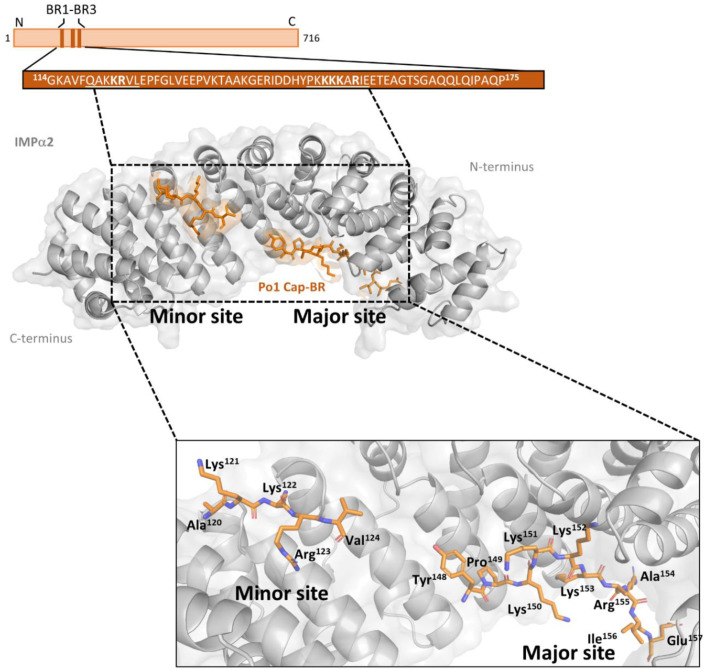
**Crystal structure of AAV Po1 Cap-BR in complex with IMPα2.** Schematic overview of the AAV Po1 Cap protein, structure of the AAV Po1 Cap-BR (orange sticks), and IMPα2 (grey ribbons/transparent surface) complex resolved to 2.6 Å resolution. The sequence of AAV Po1 Cap-BR bound to IMPα2 is detailed in the box (basic regions determined by sequence alignment are underlined, and residues determined to participate in the interaction are in bold). This structure has been deposited to the PDB and given the code: 8FK3.

The IMPα2:AAV Po1 Cap-BR complex was successfully crystallized [Figure 2D] via the hanging drop vapour diffusion method [61], and short rod-shaped crystals diffracted [Table 1] on the MX2 beamline at the Australian Synchrotron. The data were indexed and integrated in iMOSFLM [53], prior to merging and scaling performed in Aimless [Table 1]. Molecular replacement was performed using Phaser to solve the structure using the PDB 4OIH as a search model [62]. Rebuilding and refinement allowed for the structure to be resolved to a resolution of 2.6 Å and refined to a R_work_/R_free_ of 0.21/0.24 via iterative cycles of modelling and refinement through COOT and Phenix programs. The finalized model (Figure 3) consisted of 426 residues of IMPα2 (72–497), 15 residues of AAV Po1 Cap-BR (residues 120–124; 148–157), and 25 water molecules. The stereochemistry and other refinement statistics are presented in Table 1.

### 3.3. Binding Determinants of the AAV Po1 Cap-BR: IMPα2 Complex

**Figure 4:**
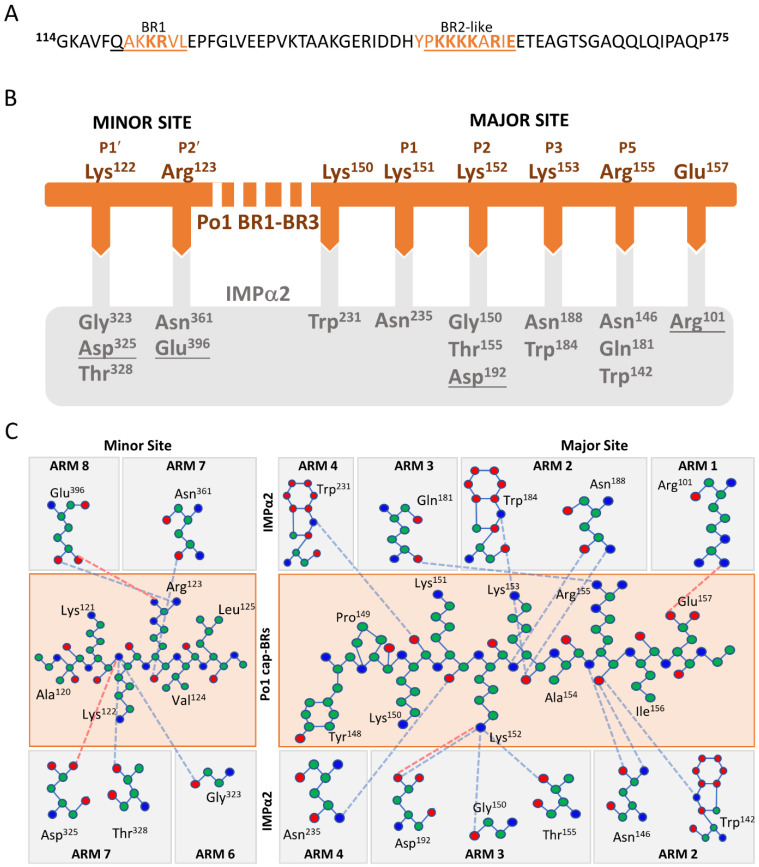
**Binding interactions of AAV Po1 Cap-BR in complex with IMPα2.** (**A**) The sequence of AAV Po1 Cap-BR used in this study, where black represents the entire sequence used to generate complexes, underlined regions show the basic regions (BR1 and BR3 indicated from the literature with BR2 indicated via alignment with AAV5), orange indicates residues that can be fitted with confidence into the IMPα2 crystal structure, and residues in bold have direct binding interactions with IMPα2. (**B**) Simplified representation of IMPα2 and AAV Po1 Cap-BR binding interactions. The AAV Po1 Cap-BR (orange line) residues bound to IMPα2 (grey box) as indicated through complementary arrows. Salt bridges are indicated via underlined IMPα2 residues, and non-underlined residues indicate hydrogen bonds. (**C**) A schematic overview of the interactions occurring through IMPα2 residues, and corresponding ARMs (grey boxes) and AAV Po1 Cap-BR residues (orange box) in sequence order. Binding interactions are displayed through hydrogen bonds (blue dotted lines) and salt bridge interactions (red dotted lines) as indicated by the PDBsum server.

The modelled high resolution IMPα2 structure maintained the expected α-helical conformation of the ten sequential armadillo (ARM) motifs, as previously observed and described [63]. The AAV Po1 Cap-BR was observed to bind within the interaction sites typical of IMPα and NLS complexes, with both the major site of IMPα (ARM 2–4, P1–P5 sites), and minor site (ARM 6–8, P1’–P3’; sites) involved in binding interactions (Figure 3). This binding is typical of a bipartite NLS observed in other structures [64,65,66]. It was observed that the overall binding buries a total surface area of 1238.7 Å^2^ and is mediated by 16 hydrogen bonds, 12 in the major binding site and 4 in the minor, with a total of 4 salt bridges, 2 in the major binding site and 2 in the minor binding site (Figure 4B,C) (Table 2).

AAV Po1 VP1 residues of the proposed BR2 site occupy the major cargo binding site of IMPα2, in congruence with our prior publication utilizing a shorter AAV Po1 [47]. For the structure obtained with the more extensive AAV Po1 Cap-BR, the residue Lys^151^ maintains a hydrogen bond with the IMPα2 Asn^235^ residue of the P1 site (Figure 4B,C). Lys^152^ is the predominant binding determinate of the major binding site by interacting with the crucial P2 site and forming hydrogen bonds with IMPα2 Gly^150^, Thr^155^, and Asp^192^, with a classical salt bridge formed between Asp^192^ of IMPα2 and AAV Po1 Cap-BR Lys^152^ (Figure 4B,C). AAV Po1 Cap-BR Lys^153^ formed hydrogen bonds with IMPα2 Asn^188^ and Trp^184^, typical of the P3 binding site. No binding interactions were observed between IMPα2 and AAV Po1 Cap-BR within the P4 position. The P5 site did maintain a binding interaction with AAV Po1 Cap-BR Arg^155^, maintaining hydrogen bonds with the IMPα2 residues Asn^146^, Gln^181^, and Trp^142^ (Figure 4B,C).

In the minor cargo binding region of IMPα2, the expected canonical ‘KR’ motif of the AAV Po1 VP1 BR1 site can be resolved, indicating that the VP1 capsid protein indeed interacts with the host IMPα via a bipartite NLS. The two residues Lys^122^ and Arg^123^ form hydrogen bonds with IMPα2 P1′ and P2′ sites. With Lys^122^ interacting with Gly^323^, Thr^328^, and Asp^325^ with an additional salt bridge formed with Asp^325^, this forms the P1′ binding site. Arg^123^ occupies the P2′ site with a hydrogen bond formed with Asn^361^, and a hydrogen bond and salt bridge connecting to Glu^396^ (Figure 4B,C). These interactions with the ‘KR’ residues of AAV Po1 Cap-BR are typical of other classical bipartite NLS structures involving IMPα2 [64,65,66,67].

### 3.4. Biochemical Assay Demonstrates AAV Po1 VP1 Has Specificity for IMPα Interaction over IMPβ Indicating Use of Classical Nuclear Import Pathway

**Figure 5:**
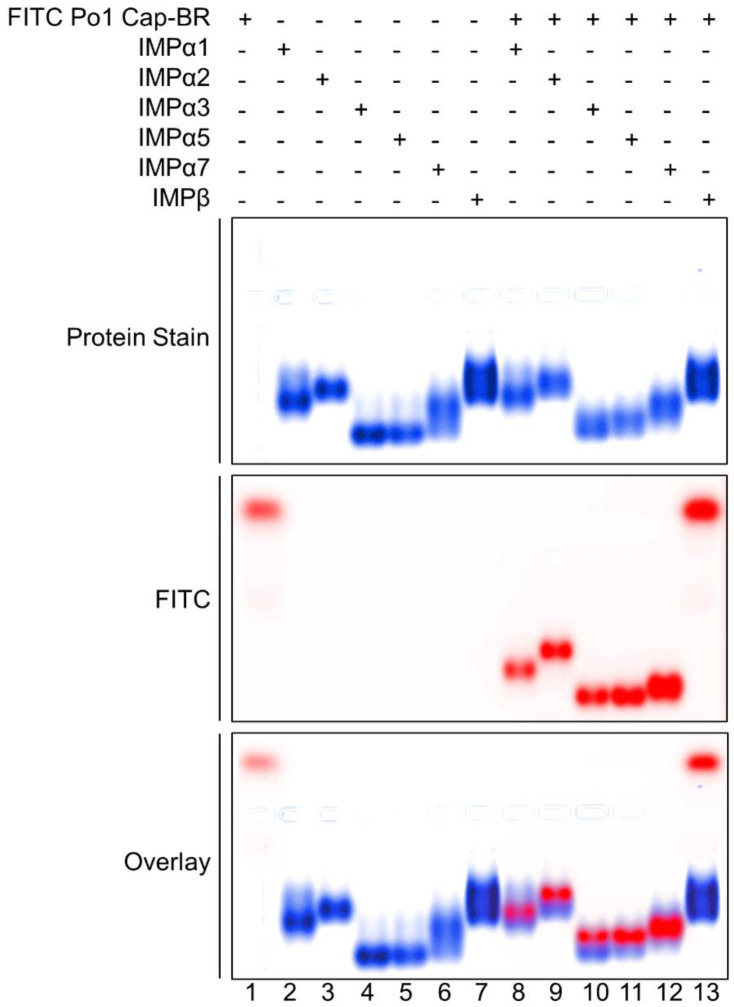
**EMSA showing AAV Po1 Cap-BR with importin isoforms.** EMSA showing AAV Po1 Cap-BR with ΔIBB-IMPα isoforms spanning members from each of the three subfamilies (SF1: IMPα1/2; SF2: IMPα3; SF3: IMPα5/7). The AAV Po1 Cap-BR peptide spans residues 119–158, and contains an FITC and Ahx linker (middle panel, shown in red). Proteins were stained using Coomassie blue stain (top panel; blue), and the overlay is represented in the bottom panel, where FITC peptide (red) overlays with protein (blue). EMSA results are representative of three independent experiments.

EMSAs were utilized to assess AAV Po1 Cap-BR interactions with importin isoforms, including IMPβ. Through three independent experiments, it was determined that AAV Po1 Cap-BR could bind with IMPα across the three subfamilies SF1 (α1, α2); SF2 (α3); and SF3 (α5, α7), but did not bind with IMPβ (Figure 5). These data show an obvious preference for binding IMPα, consistent with a classical nuclear import pathway whereby the NLS binds to the IMPα adapter, which in turn binds IMPβ to form a complex prior to translocating to the nucleus. A lack of binding to IMPβ (Figure 5) suggests that IMPβ cannot act as a direct binder with AAV Po1 VP1 for non-classical localization, but rather needs IMPα for translocation into the nucleus.

### 3.5. Biochemical Assessment of Binding Affinities Supports the Model of an AAV Po1 VP1 Bipartite NLS

**Figure 6:**
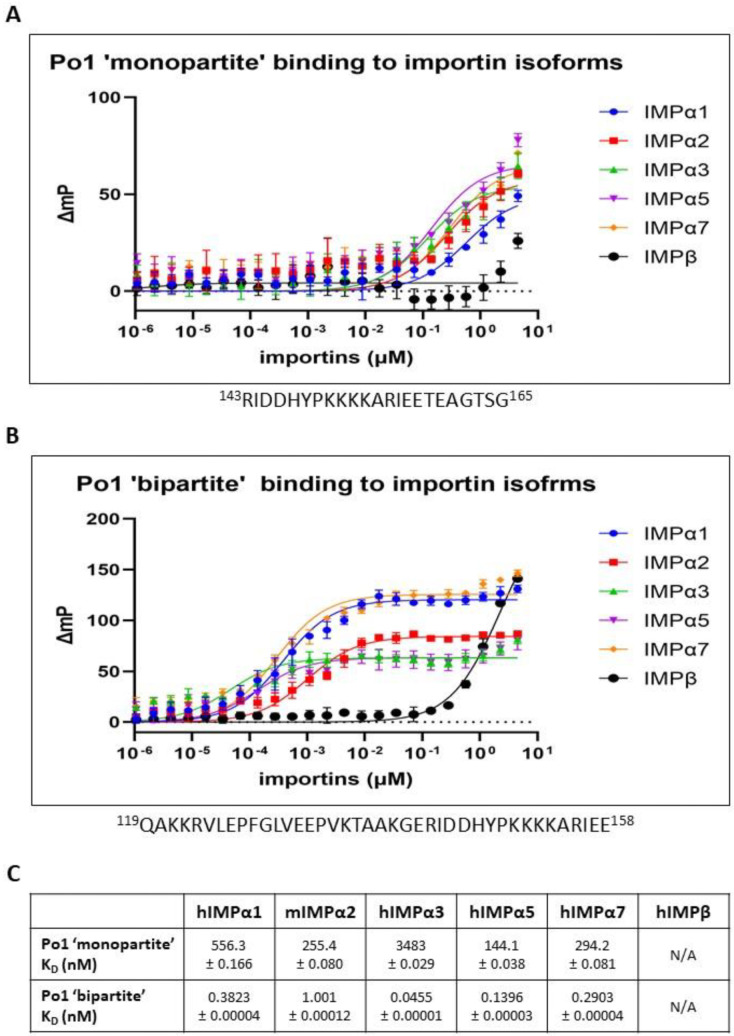
**AAV Po1 Cap-BR demonstrates a greater binding affinity for importin isoforms than monopartite AAV Po1 Cap-NLS.** (**A**) FP assay measuring the direct binding between the monopartite AAV Po1 Cap-NLS and respective importin isoforms. Weak binding was observed with IMPα1 (556.3 nM), IMPα2 (255.4 nM), IMPα3 (3483 nM), IMPα5 (144.1 nM), and IMPα7 (294.2 nM). Binding with IMPβ was so low that an accurate K_D_ was not able to be calculated. (**B**) FP assay measuring the direct binding between the bipartite AAV Po1 Cap-BR and respective importin isoforms. Strong binding in the 45 pM–1 nM range was observed with IMPα1 (382.3 pM), IMPα2 (1.001 nM), IMPα3 (45.5 pM), IMPα5 (139.6 pM), and IMPα7 (290.3 pM). Much weaker binding was observed with IMPβ, and the binding affinity was difficult to calculate as saturation was not obtained. Error bars for panels A and B were calculated using the standard deviation from the mean of 3 independent experiments. **(C)** K_D_ of direct binding between importin isoforms and AAV Po1 Cap peptides in nM concentration. K_D_ difficult to calculate due to low binding is represented by N/A. The error was the standard error of the mean.

FP assays were undertaken to not only determine qualitatively the capacity for AAV Po1 Cap-BR to be bound by different importin isoforms (including IMPβ), but also to quantitatively measure the differences in binding affinity between AAV Po1 VP1 monopartite NLS [47] and the bipartite NLS revealed in this study. We observed a clear difference in the strength of binding between monopartite and bipartite NLSs (Figure 6). AAV Po1 Cap bipartite NLS showed a very strong binding affinity for all importin-α isoforms, with binding occurring in the low nM range, but only very weak binding with IMPβ (Figure 6B). In contrast, the monopartite AAV Po1 Cap-NLS showed severely reduced binding with all IMPα isoforms compared to AAV Po1 Cap-BR, and no detectable binding to IMPβ (Figure 6A).

## 4. Discussion

Here we describe the structure of AAV Po1 Cap-BR bound to IMPα, identifying a bipartite binding mechanism. This bipartite binding greatly increases the AAV Po1 VP1 affinity for binding host IMPα proteins when compared to the monopartite AAV Po1 Cap-NLS [47]. This bipartite NLS extends from the common BR1 observed across almost all AAVs to a BR2 further downstream, rather than the typical BR2 found on other AAVs. Due to the manner in which the capsid proteins are translated, BR1 and BR2 are present on VP1 whilst BR1 is not found on VP2. This is of particular note considering that the N-terminus (and its cohort of basic regions) of VP1 is suspected of driving the virion into the nucleus due to exposure through the virion pore [42,68,69]. Our data support this notion, considering the overall difference in the strength of binding found between monopartite AAV Po1 Cap-NLS, bipartite AAV Po1 Cap-BR, and respective IMPα isoforms. Considering VP2 would only contain the weaker binding monopartite NLS (BR2) whilst VP1 holds the bipartite NLS (BR1 and BR2), it can be reasoned that VP1 would be more efficient in mediating nuclear localization. VP3 lacks all basic regions, and in the absence of VP1 and/or 2, does not accumulate in the nucleus [70,71].

BR1 is common across all AAV VP1 proteins including those lacking the typically expected BR2 and BR3. This information paired with the evidence that the inclusion of AAV Po1 VP1 BR1 in the bipartite NLS leads to stronger binding with IMPα than without, gives valuable insight into the way in which BR1 of other AAVs may be either assisting in driving VP1 nuclear localization, or even possibly be driving binding with importin proteins.

Whether weaker interactions between IMPα and the VP2 monopartite NLS also assist the function of VP1 and contribute to nuclear translocation of the virion remains to be directly investigated. Future nuclear translocation studies should be performed using recombinant AAV virions, wherein VP1 possesses a normal BR1 and BR2, whilst VP2 has a mutated BR2 to determine if this monopartite VP2 NLS contributes to transduction at all.

## Figures and Tables

**Table 1:** Data collection and refinement statistics for the structure of importin-α in complex with AAV Po1 basic regions 1–3.

AAV Po1 Cap-BR and IMPα2 (PDB Code 8FK3)	
Data Collection	
Wavelength	0.9537
Data-collection temperature (K)	298
Detector Type	ADSC Quantum 210r
Detector	CCD
Resolution range (Å); (^o^)	19.97–2.60
Space group	P 21 21 21
Unit cell (Å)	78.15 Å 89.46 Å 99.39 Å
Total reflections	83,199 (10,417)
Unique reflections	21,468 (2616)
Multiplicity	3.9 (4.0)
Completeness (%)	97.9 (99.1)
Mean I/σ (I)	9.6 (2.5)
Wilson B-factor Å^2^	41.351
R_pim_	0.064 (0.502)
**Refinement**	
R_work_	0.2119 (0.2990)
R_free_	0.2488 (0.3320)
No. of non-hydrogen atoms	3304
Macromolecules	3279
Solvent	25
Protein residues	441
Bond length r.m.s.d (Å)	0.003
Bond angle r.m.s.d (^o^)	0.53
Ramachandran favoured (%)	97.70
Ramachandran allowed (%)	2.30
Ramachandran outliers (%)	0.00

**Table 2:** Hydrogen bond and salt bridge interactions.

AAV Po1 Cap-BR Hydrogen Bond and Salt Bridge Interactions with IMPα2	
**Hydrogen bonds**	
**IMPα2**	**AAV Po1 Cap-BR**
TRP 142 [HH12]	ARG 46 [O]
ASN 146 [O]	ARG 46 [O]
ASN 146 [ND2]	ARG 46 [O]
GLY 150 [O]	LYS 43 [NZ]
THR 155 [OG1]	LYS 43 [NZ]
GLN 181 [OE1]	ARG 46 [NH1]
TRP 184 [NE1]	LYS 44 [O]
ASN 188 [OD1]	LYS 44 [N]
ASN 188 [ND2]	LYS 44 [O]
ASP 192 [OD1]	LYS 43 [NZ]
TRP 231 [NE1]	LYS 41 [O]
ASN 235 [ND2]	LYS 42 [O]
GLY 323 [O]	LYS 14 [NZ]
THR 328 [OG1]	LYS 14 [NZ]
ASN 361 [OD1]	ARG 15 [N]
GLU 396 [OE1]	ARG 15 [NH2]
**Salt Bridges**	
**IMPα2**	**AAV Po1 Cap-BR**
ARG 101 [NH1]	GLU 48 [OE1]
ASP 192 [OD1]	LYS 43 [NZ]
ASP 325 [OD1]	LYS 14 [NZ]
GLU 396 [OE1]	ARG 15 [NH2]

## Data Availability

Data are available upon request via the corresponding author.
